# An Ishmaelite

**Published:** 1888-07-21

**Authors:** Leslie Keith

**Affiliations:** Author of "The Chilcotes," "Uncle Bob's Niece," "East and West," etc.


### An Ishmaelite.

By Leslie Keith,

Author of "The Chilcotes," "Uncle Bob's Niece," "East and West," etc.

(Continued from p. 250.)

### Chapter XV.

The autumn, which saw Arthur in London, sharing the gloom of the big empty house in Portland Place with his uncle, found Nellie paying a round of country visits with her husband and child.

The visits were to various members of the Bellynge family. "The family," as Richard Bellynge was in the habit of calling his people when talking to his wife. Nellie used to feel a little sore on this point; Richard would talk as if she ought to feel very much flattered by the welcome of this big clan. He took her to all the dances and dinners given by his numerous sisters and cousins in the season, and was rather peremptory in insisting that she should keep up friendly relations with them all, while, if he did not absolutely discourage visits to her own kindred, he showed plainly enough that he took scant interest in hearing of them.

Poor little Nellie! The old feeling of being in disgrace frequently haunted her still. Her husband was generous enough to her. She had a smart little victoria in which to drive about and see the relations, who were very condescending and kind, and usually frightened her into a stupid silence. The House in Pont Street was always hospitably thrown open, but it was Bellynge's, and the approved Bellynge set trooped into it, and managed its entertainments, and patronised the shy hostess, who was voted "pretty enough, and a harmless little thing, but nothing in her, you know."

When Nellie wanted to see Arthur, she had him to lunch while Richard was at the bank, and the two could be sure of being alone.

The brothers-in-law did not "hit it off," and Arthur did not hide the fact that the Bellynge clique with their "little language" bored him.

Arthur had a very strong affection for Nellie, however, and a kind of protecting tenderness for her, though she was a year or two older than he. He went often to see her before the tide of visitors set in, but at the first rat-tat at the door he would make off.

"I can't stand your fine ladies," he said, "they always put my back up."

"It is Lady Maria," said Nellie faintly, hearing the familiar voice in the hall.

"Then I'm off," said Arthur, jumping up with a laugh. "If I remain she will want to form me too. Don't let her devour you, Nellie."

On that last visit, when Nellie and her husband were living incognito, as it were, in their dismantled house, before going off to visit a new cousin or aunt, Arthur had promised to write to his sister. The letter found her in a highland castle, which the head of the family, Uncle Bromfield-Bellynge, had taken for the season. She felt rather lost among its rugged splendours, its gay company, and could not talk the fashionable slang of the other ladies, nor flirt openly with the men as they did. She was happiest when in the nursery with her baby. Her husband was out all day on the moors, and she saw little of him.

Arthur's letter, then, came to her as a voice from home—her own home, where she was not a mere sister-in-law, and a stupid little thing, to be coaxed, and managed, and drilled. She kissed the lines that Arthur wrote out of a full heart, and cried over them in secret. Arthur told her his disappointment about Fred, and how his dreams of a new Utopia across the ocean had all ended in a poor little back street in London, where Fred had insisted on burying himself.

Fred in London! Nellie's heart gave great throbs as she thought of him there, and the colour came deep, deep into her cheeks.

She was staring absently out of the window of her bedroom, where she had retired, with the letter spread upon her lap; her thoughts had taken a leap backwards over the years, and she was living again in her girlhood, with its brief glimpse of happiness and its long, long night of sorrow.

"Shall I ever see him again?" she wondered, her pulses throbbing in time to her crowding emotions. It was while she was sitting there, looking out with unseeing eyes upon the sunny world, that her husband startled her by suddenly entering the room.

He flung himself down on a sofa near her.

"How cool you look, Nell," he said. "We've had a beast of a day."

She looked up at him with frightened, wistful eyes, and tried to collect her scattered senses.

"Haven't you made a good bag?" she said, and she did her best to listen to his account of the day, and to sympathise with its misadventures.

"Who is your correspondent?" he asked at last, his eye falling on the letter on her lap.

"It is from Arthur," she said. She hastily slid the letter into her pocket with a blush.

"Where is Arthur? The Black Castle people expected him for the 12th, but Fraser tells me he didn't turn up."

"He is in London."

"In London—in this weather?" Richard Bellynge lifted his eyebrows. "What new craze for martyrdom is keeping him there?"

"Uncle Joseph is there," Nellie began falteringly; and then, obeying a sudden impulse, she rose, and going up to him, sat down on the edge of the sofa where her husband lay. A great longing for sympathy had taken possession of her.

"Richard," she said, laying her hand timidly on his, "Arthur is staying in town because—because of Fred ——"

"What!" said Bellynge with a frown. "Is that scamp let loose again? And I suppose Arthur is taking up the cudgels in his defence, and making him out to be an injured, persecuted saint ——?"

The colour deepened on Nellie's cheeks, and she withdrew her hand. "Arthur is my brother," she said coldly.

Richard Bellynge loved his little wife in his own fashion, though he did not always understand her. He sat up and put an arm round her waist, drawing her nearer to him.

"I didn't mean to hurt you, Nellie," he said; "but Arthur's nonsense always stirs my bile. He expects to convert the world with his committees for the promotion of virtue, and his white-ribbon leagues, but that pale quixotic folly won't go down in these days. He ought to have been born a century or two earlier."

"You never knew Fred," said Nellie with a flicker of resistance, "you can't be expected to understand what Arthur feels"—what I feel, she would have added, but she lacked the courage.

"I never knew him, certainly, and i've no particular wish to begin the acquaintance now. Your cousin may have been everything that was delightful Nellie, but he made a mess of his life, and he has got to abide by the consequences. Arthur can't present him with a new set of morals and launch him on the world again. Society draws the line, and rightly enough, at released gaol-birds. It is lax enough, heaven knows, but it stops short at that."

"You are hard, Richard," said Nellie, with a lump in her throat.

"No, my dear, I am only practical, If your cousin wanted to keep his place he ought to have thought twice before he took to forging anybody's signature. Any sensible woman would see it in that light."

"You ought to have married one of your cousins, Richard," said Nellie, with a tinge of bitterness, "they are sensible enough."

"I am quite satisfied with my little wife," he said with perfect good humour," and I mean to look well after her. There, that's the dressing bell, run and make yourself smart. And look here, Nellie," he called after her, "we'll wash our dirty linen in private, you know; I won't have that story revived to be talked over and discussed among the relations. It is a mercy after all Arthur didn't turn up at Black Castle, or we should have been publicly invited to take the returned prodigal by the hand."

"I am not likely to speak of Fred to your sisters," said Nellie proudly, "you needn't be afraid, Richard."

She went away with an unhealed soreness in her heart; she was expected to deck herself and be gay, and smiling, and pretty, and to crush down her better feelings, and to hide them under the smartest frock in her wardrobe. She knew Richard would watch her, and for the first time in her married life she felt a dull, creeping resentment against him.

"Does marriage always mean this complete severance—this sharp division of oneself"—she wondered in her bewilderment—"this surrender of all the old tender affections and traditions?"

If Richard Bellynge had taken the trouble to understand his wife a little better, he would have spared himself and Nellie many days of needless pain. He might have won her allegiance so easily—there never was a creature so easily won as simple, silly Nellie. But he only hardened her, and frightened her into taking the first step in the path of alienation.

She had never before disobeyed her husband, but she knew she was traversing his express wishes when she wrote to Fred. He had not said, indeed, in so many words "you must neither write nor see him," and that sophistry may have helped to soothe her uneasy conscience.

Nellie put on her smartest dress and her finest jewels, and was so gay and bright during dinner that Lady Maria decided that something might be made of the stupid little thing after all; but when her husband had gone to the billiard-room she slipped upstairs and wrote in trembling haste a little note which she locked away in her dressing-case.

It did not go with the household correspondence in the mail-bag—it lay indeed in its secret hiding-place for a whole day, till Nellie found some excuse to drive to the village and post it there with her own hand.

Nobody saw the little message go fluttering into the open slit whence no second thoughts could recover it. The little bit of diplomacy was quite safely managed, and at the cost of nothing but a wound to Nellie's conscience, and a growing dread of discovery that made her tremble in her husband's presence and escape from it when she could.

Arthur would have said she had done well to write the letter, but he would have had her pen it under her husband's eye, and with his consent. Nellie characteristically took what seemed the easier way, and found it a path of thorns.

The trembling, half-incoherent note went upon its way like other letters, and was duly delivered in Gillespie Street along with the Proudie correspondence. There was a letter from an uncle in Australia, not immaculate as to spelling, but hearty in its tone and full of good tidings, which caused the family much innocent gratification.

"I always said Peter would get on," said Mr. Proudie, rubbing his hands in his satisfaction. "Peter was always a well-doing chap."

"You'll be coming in for a fortune now," said the cripple, looking at Delia with hungry jealousy, "And setting up to be heiresses——."

"Fortune? Nonsense!" said Mr. Proudie easily. "Peter will be for marrying, himself, now that he's got the siller, and if he gets as good a wife as mine, I won't cry pity on him." He gave his helpmeet a hearty salute as he tied on his apron and bustled out of the room.

Delia was the first to spy out the little letter lying under the bulky Australian scrawl, and she picked it up and ran downstairs with it.

Barbara was standing at the desk, and Fred, at the opposite limits of the shop, was trying to find a corner for a new set of volumes which the lavish Arthur had sent from town.

Delia came dancing into the shop.

"Such news, Barbara!" she cried: "A letter from Uncle Peter that makes you see money-bags and hear the chink of gold, only to read of it. Uncle Peter is quite a patriarch, with his flocks, and his herds, and his menservants. I don't know about maidservants. If we get such another letter from him, we shall have to pack up the library, and go off among the sheep."

Fred looked up hastily.

"Oh, I beg your pardon, Mr. Eastgate," said Delia, catching the look. "This is a letter for you, which I came to give you. I hope it brings you good news, too."

It was the first letter he had received since he became a member of the Proudie household. The sisters knew this, very likely; but they were far too delicate-minded to watch him while he read it.

Let Barbara repudiate the title as she might, she and her young sister were ladies in the best sense of that misused word.

Fred received the letter from Delia with a dull surprise. Who could be writing to him excepting Arthur? He had not a single acquaintance in the world, where he had once had so many.

"Help me to tie this parcel, Del," said Barbara. "My clumsy fingers can't manage it."

"Oh, you must seal it," said the practical Del. "That gum is too weak to hold it. Wait one moment, and I will light the gas."

She struck a match, and, reaching up, lit a jet of gas that hung down near the desk. They were both busy over the little matter of arranging the bulky parcel when Fred came up to them.

His face was white; and the hand in which he held out the letter shook as he reached it across the desk.

"Miss Delia," he said, "you have a light. Will you please burn this?"

She looked up surprised, scarcely understanding him for a moment.

"Burn your letter?" she said.

He still held it out mutely, and she took it from him and lifted one corner of it to the flame. Barbara looked on for an instant, and then she turned away and busied herself with the papers and ledgers on the desk.

The letter was written on thick paper, and it burned slowly, smouldering and shrivelling into black tinder. Delia deftly changed her hold of the paper till only a morsel remained unconsumed; she let that flutter downwards and put her foot on it to extinguish the flame.

"It is all gone," she said.

"Thank-you," said Fred in a low voice, and turned away.

Some fragments of the blackened tinder fell on the book over which Barbara bent. She did not disturb them, and when she had ended her consultation of the ledger, she shut it more gently than usual, and the airy fragments of tinder remained to witness to that curious little scene.

Fred gave no explanation of his sudden emotion, and indeed, fewer words than usual passed between the fellow-workers. It was a Saturday, and a busy day, since many clients came to take out their portion of holiday literature; there were also some customers for letter-paper and envelopes, and for the weekly issues of various papers and periodicals.

When the girls were alone in their room at night, Delia said suddenly:

"Wasn't that a queer thing of Mr. Eastgate to ask me to do this morning? He might have torn his letter up if he didn't want to keep it. If I had said anything about it upstairs, what a nice little mystery George Tucker would have made out of it!"

"You didn't, Delia?"

"No," said Delia, yawning, "I didn't want to give that little viper any more chances of stinging."

It was such a long time before Barbara spoke again that Delia, who had got into bed, was half asleep.

"Perhaps," she said, "he was burning his boats."

"Boats?" said Del, in a drowsy voice. "It was a letter.'

Barbara smiled, and then she got up and blew out the candle.

"It is no business of mine," she said to herself.

*(To be continued.)*

